# Linking dynamic cerebral autoregulation and inhibitory executive function: Role of modifiable behaviours

**DOI:** 10.1113/EP092669

**Published:** 2026-02-17

**Authors:** Hayato Tsukamoto, Damian M. Bailey

**Affiliations:** ^1^ Faculty of Sport Sciences Waseda University Saitama Japan; ^2^ Neurovascular Research Laboratory, Faculty of Life Sciences and Education University of South Wales Pontypridd UK

**Keywords:** aerobic exercise, breakfast, cerebral blood flow, cognitive function, glycaemic control, oxidative stress

## Abstract

Dynamic cerebral autoregulation (dCA) stabilises cerebral blood flow (CBF) against rapid fluctuations in perfusion pressure and may serve as a key physiological mediator of cognitive function. Inhibitory executive function, a core domain essential for goal‐directed behaviour, is influenced by modifiable lifestyle factors such as physical activity and dietary behaviours, yet the mechanisms linking these behaviours to inhibitory executive function remain poorly understood. This review synthesises evidence examining the relationship between dCA and inhibitory executive function in response to feasible behavioural interventions. Acute aerobic exercise (AE) preserves dCA and enhances inhibitory control, whereas exhaustive AE impairs both. Similarly, dietary behaviours such as breakfast skipping or post‐prandial hyperglycaemia acutely reduce dCA and inhibitory executive performance, suggesting a potential mechanistic link between vascular regulation and cognitive outcomes in short‐term contexts. Long‐term effects of physical activity on the dCA–inhibitory executive function relationship are less clear, likely reflecting complex interactions among vascular, metabolic and neural systems. While causal pathways remain to be fully elucidated, impaired dCA may contribute to reduced inhibitory control, highlighting its potential role as a physiological mediator. Conversely, maintaining intact dCA may be critical for supporting cognitive performance. Understanding how modifiable behaviours influence both dCA and inhibitory control provides novel insights into the physiological mechanisms underlying cognitive health. These findings could inform lifestyle‐based strategies aimed at optimising inhibitory executive function and preserving cognitive performance across the lifespan.

## Abstract (highlights)

- **What is the topic of this review?**
  This review explores the dynamic relationship between dynamic cerebral autoregulation (dCA) and inhibitory executive function, emphasising how modifiable behaviours – particularly physical activity and dietary patterns – affect both.
- **What advances does it highlight?**
  Aerobic exercise enhances inhibitory executive function while maintaining dCA, whereas exhaustive exercise impairs both. Similarly, dietary behaviours such as breakfast skipping and post‐prandial hyperglycaemia compromise dCA and inhibitory executive function. These findings underscore a mechanistic link between lifestyle behaviours, cerebrovascular regulation and higher‐order cognitive function.

## 1 INTRODUCTION

### 1.1 The importance of inhibitory executive function in humans

The development of the prefrontal cortex (PFC) is a hallmark of the human brain compared with other species (Fuster, 2002). A key PFC function is executive function (Diamond, 2013), encompassing cognitive processes such as shifting mental sets, monitoring/updating working memory and inhibitory control (Miyake et al., 2000) (Figure 1). Inhibitory executive processes underpin everyday decision‐making (Funahashi, 2001) and decline with ageing, a non‐modifiable factor (Buckner, 2004). This decline is associated with mild cognitive impairment and increased dementia risk (Kirova et al., 2015). Modifiable behaviours, such as physical activity and dietary intake, can influence inhibitory executive function, but effects vary widely (see below), highlighting the need to understand the physiological mechanisms underlying these outcomes. Physical activity (energy expenditure) and dietary intake (energy consumption) represent influential and widely accessible lifestyle behaviours throughout the lifespan.

**FIGURE 1:**
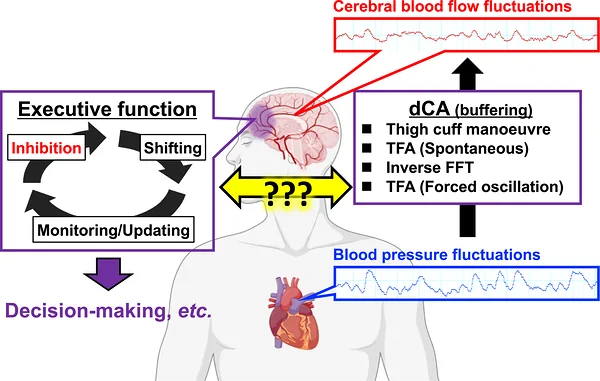
Schematic representation of executive function and dynamic cerebral autoregulation (dCA). Executive function, supported by prefrontal cortex activation, encompasses shifting mental sets, updating working memory, and inhibitory control. dCA maintains relatively stable cerebral blood flow against rapid perfusion pressure fluctuations and can be assessed using methods such as the thigh cuff manoeuvre, transfer function analysis (TFA) of spontaneous and/or forced oscillations, and inverse fast Fourier transform (FFT).

### 1.2 Cerebral blood flow and cerebral autoregulation

The human brain (1.179–1.621 kg) is a high‐energy organ with a mass‐specific resting metabolic rate of 1008 kJ/kg/day (Molina & DiMaio, 2012; Pontzer et al., 2021). Although it primarily relies on glucose/glycogen, stored glycogen is limited (≤7 µmol/g) in healthy individuals (Öz et al., 2015). Despite the blood–brain barrier, the brain continuously requires circulating energy substrates, including glucose (Wasserman, 2009), lactate (Hashimoto et al., 2021), ketone bodies (Pellerin, 2010) and oxygen (Bailey et al., 2017). Thus, cerebral blood flow (CBF) regulation is critical for supporting neuronal activity and maintaining inhibitory executive function.

CBF is regulated by complex physiological mechanisms. Among them, Lassen (1959) first described cerebral autoregulation (CA) as the maintenance of steady state CBF across mean arterial pressure (MAP) ranges of 60 to 150 mmHg, despite pharmacological hypo‐ or hypertension. Tiecks et al. (1995) demonstrated that during propofol anaesthesia, gradual increases in arterial blood pressure (ABP) do not alter middle cerebral artery blood velocity (MCAv), whereas high‐dose isoflurane impairs CA – this concept is termed ‘static’ CA.

Although the classical Lassen curve has provided a foundational framework for understanding CA (Lassen, 1959), more recent work has challenged the concept of a broad pressure‐independent plateau. For example, Brassard et al. (2021) suggest that CBF may be more pressure‐passive than traditionally assumed, particularly within the physiological range of ABP. Moreover, the transient response of CBF to rapid changes in ABP reveals dynamic, time‐dependent regulation rather than steady‐state independence, highlighting the importance of considering CA as a dynamic, frequency‐dependent process. CBF fluctuates with rapid ABP changes (Aaslid et al., 1989; Panerai et al., 2023). Dynamic CA (dCA) buffers these fluctuations, maintaining cerebral bioenergetic function and corresponding homeostasis (Aaslid et al., 1989; Panerai et al., 2023; Tiecks et al., 1995). This perspective has important implications for interpreting both static and dynamic measures of CA. Impaired dCA is associated with stroke risk (Chi et al., 2018).

Because of its high temporal resolution, transcranial Doppler is particularly effective for evaluating dCA, as it allows continuous measurement of cerebral blood velocity (e.g., MCAv) (Brassard et al., 2023). Traditionally, dCA is commonly assessed via the thigh cuff manoeuvre or transfer function analysis (TFA) of MAP and MCAv. For example, MAP and mean MCAv (MCAv_mean_) rapidly decrease immediately after the sudden release of inflated thigh cuffs, and dCA functions to recover CBF from 1.0 to 3.5 s after thigh cuff release (Tiecks et al., 1995). In this context, the slope of cerebrovascular resistance index recovery (MAP/MCAv_mean_) following cuff release provides an index of the rate of regulation (RoR), reflecting the effectiveness of dynamic cerebral autoregulation (dCA) in counteracting passive changes in perfusion pressure (Aaslid et al., 1989). During the thigh‐cuff manoeuvre, a reduced RoR is interpreted as impaired dCA. Meanwhile, given that ABP spontaneously fluctuates and dCA is a frequency‐dependent phenomenon, TFA estimates coherence, gain and phase across frequency bands. It is known that very low (VLF, 0.02–0.07 Hz, 50 to 14.3 s cycles), low (LF, 0.07–0.20 Hz, 14.3 to 5 s cycles), and high (HF, 0.20–0.50 Hz, 5 to 2 s cycles) frequency bands reflect dCA (Panerai et al., 2023; Zhang et al., 1998), while the ultra‐low frequency (<0.02 Hz, >50 s cycles) band reflects static CA (Brassard et al., 2021). The coherence indicates the fraction of MCAv_mean_ power that is linearly related to the MAP power at each frequency. Increases in gain or decreases in phase indicate impaired dCA, though discrepancies between indices can occur, resolvable via step‐response analysis and the autoregulation index (ARI) (Panerai et al., 2023). A further limitation of TFA applied to spontaneous fluctuations is the typically low coherence between ABP and CBF signals. Forced oscillations via oscillatory lower body negative pressure or repeated sit–stand or squat manoeuvres improve coherence but may introduce confounding physiological changes (Panerai et al., 2023). These analytical approaches provide complementary rather than redundant information, and apparent discrepancies across studies may reflect methodological differences rather than true physiological disagreement.

CBF is modulated not only by dCA but also by cerebrovascular CO_2_ reactivity and neurovascular coupling (NVC) (Ainslie & Duffin, 2009; Phillips et al., 2016). Similarly, CA is influenced by physiological alterations in myogenic, metabolic and neurogenic components (Brassard et al., 2023; Claassen et al., 2021). For example, RoR increases during hyperventilation‐induced hypocapnia and decreases with 5% CO_2_ inhalation (Aaslid et al., 1989). Cognitive activity elevates CBF, attenuating dCA during spontaneous ABP fluctuations (Ladthavorlaphatt et al., 2024; Ogoh et al., 2018). Thus, although the precise contribution of ‘ceiling’ or ‘floor’ effects of vascular tone to observed dCA responses cannot be functionally delineated, these observations support that different vasomotor stimuli influence dCA (Figure 2).

**FIGURE 2:**
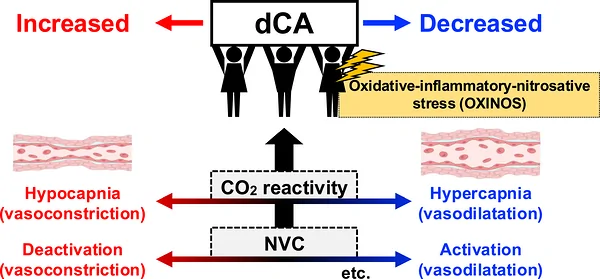
The interaction between dynamic cerebral autoregulation (dCA) and cerebrovascular CO_2_ reactivity or neurovascular coupling (NVC). Cerebrovasomotor tone, as well as oxidative–inflammatory–nitrosative stress (OXINOS), is regarded as a major factor regulating dCA.

This review aims to synthesise current evidence on how aerobic exercise (AE, energy expenditure) and diet (energy consumption), as modifiable lifestyle behaviours, influence inhibitory executive function and dCA in young individuals, while also examining the relationship between these processes in the context of brain health.

## 2 ENERGY EXPENDITURE

### 2.1 Aerobic exercise

Among lifestyle behaviours, physical activity plays a central role in shaping systemic physiological regulation. Physical activity, as a modifiable behaviour, exerts widespread effects on cardiovascular, metabolic and neural systems. In this context, it is well established that regular physical activity provides broad health benefits, including the preservation of brain function (Boa Sorte Silva et al., 2024; Garber et al., 2011; Pedersen & Saltin, 2015). However, inappropriate modality, intensity, duration or frequency of exercise can have detrimental consequences for brain health, highlighting exercise as a double‐edged sword (see below). For example, insufficient intensity may fail to elicit meaningful neuroplastic adaptations, whereas excessive workload or exhaustive (i.e., all‐out effort) exercise may induce oxidative stress, impair CBF regulation, or compromise blood–brain barrier integrity. It should be noted that exercise workload is determined by both intensity and duration and is closely associated with perceived exertion; according to the Borg 6–20 scale, the rating of perceived exertion (RPE) is generally classified as very light (≤11), moderate (12–14), vigorous (15–17) and all‐out effort (≥18) (Borg, 1982). Thus, defining the ‘optimal dose’ of exercise remains an important challenge in neuroscience and physiology.

Voss et al. (2020) proposed that the magnitude of acute cognitive responses to exercise may serve as a partial predictor of longer‐term outcomes, but acute responses and chronic adaptations share both overlaps and divergences. Chronic adaptations are more likely to involve structural and functional brain changes, including increased grey matter volume (Colcombe et al., 2006), enhanced white matter integrity (Bashir et al., 2021), strengthened functional connectivity (Voss et al., 2020) and improved regulation of CBF (Bailey et al., 2013). These adaptations are also modulated by systemic factors such as the release of circulating exerkines, upregulation of neurotrophic factors, angiogenesis and improved redox homeostasis (Calverley et al., 2020; Hashimoto et al., 2021). Importantly, these long‐term changes likely build upon – but are not fully explained by – the transient responses observed after a single exercise bout.

Notably, even the acute physiological changes induced by a single exercise session are highly complex and multifactorial. They involve interactions between NVC, dCA, substrate metabolism and neurotransmitter release, all of which can transiently alter inhibitory executive function. The precise mechanisms underlying these acute effects remain incompletely understood and are the focus of ongoing research aimed at disentangling the dose‐dependent and context‐specific impacts of exercise on brain health.

### 2.2 A single bout of aerobic exercise and inhibitory executive function

A recent meta‐analysis confirmed that a single bout of AE improves inhibitory control, although the magnitude of this effect varies across populations (Chang et al., 2025). With respect to cognitive function in young, healthy individuals, even very light‐intensity AE (≤50% maximum heart rate) performed for 10 min can enhance inhibitory executive function (Byun et al., 2014). However, these improvements are small in effect size and transient, with no sustained benefit (Chang et al., 2012; Tsukamoto et al., 2017). Increasing AE intensity appears to be a useful strategy for eliciting greater improvements in young, healthy participants (Lucas et al., 2012; Tsukamoto et al., 2017). Indeed, meta‐analytic evidence confirms that moderate‐intensity AE produces larger effects than light‐intensity AE (McMorris & Hale, 2012).

Interestingly, an inverted‐U relationship between AE intensity and inhibitory executive function has been reported (McMorris & Hale, 2012). For example, executive function declines during cycling at 80% heart rate reserve within 40 min (RPE 18) (Wang et al., 2013), and running at 75% maximal oxygen uptake (${\dot{V}}_{O_{2}\max}$) for 65 min similarly impairs inhibitory executive performance in young individuals (Konishi et al., 2017). Thus, prolonged high‐intensity AE exerts negative effects. Even moderate‐intensity AE may impair decision‐making if performed for extended durations: during 3 h of cycling at ≤60% ${\dot{V}}_{O_{2}\max}$, improvements in map recognition speed and accuracy observed during the first 1–2 h disappeared after 2 h (Grego et al., 2005). Collectively, these findings indicate that the cognitive benefits of AE can be cancelled by excessive workload, whether through high‐intensity or prolonged duration.

AE can also be performed as an interval exercise. Notably, high‐intensity interval exercise (HIIE) confers cognitive benefits (Calverley et al., 2020; Lucas et al., 2015). In young, healthy participants, inhibitory control after cycling HIIE (four 4‐min bouts at 90% ${\dot{V}}_{O_{2}\mathrm{peak}}$ with 3‐min recovery at 60% ${\dot{V}}_{O_{2}\mathrm{peak}}$) exceeds that observed after volume‐matched continuous AE at 60% ${\dot{V}}_{O_{2}\mathrm{peak}}$ for 40 min (Tsukamoto et al., 2016a). Similarly, low‐volume HIIE (ten 1‐min bouts at 90% ${\dot{V}}_{O_{2}\mathrm{peak}}$ with 1‐min recovery at 30% ${\dot{V}}_{O_{2}\mathrm{peak}}$) enhances inhibitory control comparably to 40 min of moderate‐intensity AE in young, healthy participants (Sugimoto et al., 2020). Thus, repeated short bouts of high‐intensity AE can improve inhibitory executive function provided exhaustion is avoided. However, analogous to a football match, a second HIIE session performed after rest negates these benefits (Tsukamoto et al., 2016b). Excessive frequency, therefore, shifts interval training toward negative effects. In addition to this, incremental AE to exhaustion (20‐W increases for approximately 16 min) fails to improve inhibitory executive function (Sudo et al., 2017). Hence, exhaustion itself may be a key determinant of adverse effects. The acute influence of high‐intensity AE on inhibitory executive function remains under active debate (Sudo et al., 2022).

### 2.3 Cerebral blood flow regulation in acute responses to aerobic exercise

During AE, motor cortex activation increases in proportion to exercise intensity (Fontes et al., 2020). Correspondingly, MCAv rises with intensity up to moderate AE (Brugniaux et al., 2014). However, high‐intensity AE and/or prolonged moderate‐intensity AE often induces hyperventilation‐related hypocapnia, which reduces MCAv (Brugniaux et al., 2014; Ogoh et al., 2014). Although these MCAv reductions suggest impaired CBF, inhibitory executive function is nonetheless enhanced during prolonged moderate‐intensity AE, even when MCAv declines due to hyperventilation (Ogoh et al., 2014). Thus, a hyperventilation‐induced decrease in CBF may not directly account for exercise‐induced impairments in inhibitory executive function. In these circumstances, improvements in inhibitory executive function following AE are likely mediated by multiple acute mechanisms, including changes in neurotransmitter systems (Ando et al., 2024) and cerebral energy metabolism (Hashimoto et al., 2018).

CBF stability is maintained in part by dCA, which buffers cerebral perfusion against ABP fluctuations. If AE alters dCA, then exercise‐related changes in inhibitory executive function may be partially explained or predicted by dCA responsiveness. Higher intensities of AE are associated with more marked elevations in ABP (Tsukamoto et al., 2017), which may challenge the operational limits of dCA, potentially shifting the effective operating point along the autoregulatory curve. Under these conditions, dCA may not simply buffer transient ABP fluctuations around a fixed set‐point but instead operate within a moving physiological context shaped by rising perfusion pressure, metabolic demand and cerebrovascular tone. For example, in healthy individuals, Brys et al. (2003) showed that TFA gain and phase in the LF range remain unchanged during incremental AE up to moderate‐intensity, despite increased MAP variability. This indicates intact dCA during low‐ to moderate‐intensity AE even if the ABP fluctuations are altered during AE. By contrast, Ogoh et al. (2005) found that LF TFA gain is increased while there is no change in the power spectra of beat‐to‐beat variability of MAP during exhaustive AE, suggesting dCA impairment, coinciding with hyperventilation‐induced vasoconstriction. Thus, alterations in dCA cannot be attributed solely to ABP or its variability. Rather, this supports the notion that dCA may be constrained when cerebrovascular reserve is progressively exhausted or when the system is operating closer to the upper limits of the autoregulatory curve. Exhaustive AE is also linked to oxidative stress in skeletal muscle (Bailey et al., 2007) and the brain (Bailey et al., 2018, 2017), which may contribute to dCA impairment and blood–brain barrier disruption (Bailey et al., 2011). Importantly, when exhaustion is avoided, dCA appears preserved even at higher intensities. For example, during HIIE, LF TFA gain and phase remain stable, indicating intact autoregulation (Tsukamoto et al., 2019).

### 2.4 Connection between inhibitory executive function and dynamic cerebral autoregulation during aerobic exercise

During AE, dCA does not improve but continues to function as a critical homeostatic defence system. The paradox, however, is that inhibitory executive performance can improve even when autoregulatory efficiency is not enhanced. This dissociation raises the possibility that compensatory mechanisms – such as improved neural efficiency, altered neurotransmission or temporary shifts in cerebrovascular CO_2_ sensitivity – may support cognition despite unchanged dCA.

Impairments in inhibitory executive function following exhaustive AE resemble patterns of dCA dysfunction (Figure 3). Moreover, while AE activates the motor cortex and deactivates the PFC (Fontes et al., 2020), inhibitory executive function tasks themselves activate the PFC (Wagner et al., 2001), which can compromise dCA (Ladthavorlaphatt et al., 2024; Ogoh et al., 2018). Similarly, prolonged cognitive engagement reduces inhibitory executive function during moderate‐intensity AE (Tsukamoto et al., 2022). These findings suggest that both psychological and physiological stressors can impair dCA and inhibitory executive function during AE. Beyond laboratory‐based settings, the interaction between psychological and physiological stressors during aerobic exercise may have important implications for real‐world, high‐demand environments. Occupations such as firefighting or military operations often involve simultaneous physical exertion, cognitive load and emotional stress, conditions under which impairment in inhibitory executive function could adversely affect decision‐making and performance. Under these stress‐related conditions, it is possible that sympathetic activation, hyperventilation‐induced hypocapnia, elevated systemic oxidative–inflammatory–nitrosative stress (OXINOS), and ABP may collectively challenge cerebrovascular reserve (Bailey et al., 2009, 2011; Sara et al., 2022) and thus, by consequence, constrain dCA. These considerations highlight the importance of examining dCA–cognition interactions under ecologically valid, high‐stress conditions and may inform strategies to mitigate cognitive vulnerability during sustained physical and psychological stress.

**FIGURE 3:**
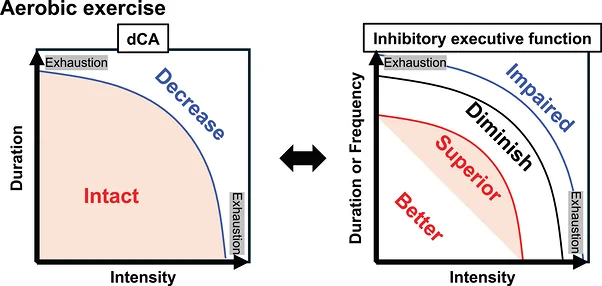
The relationship between dynamic cerebral autoregulation (dCA) and inhibitory executive function in acute response to aerobic exercise. While dCA during low‐ to moderate‐intensity exercise was not altered (intact), moderate‐intensity aerobic exercise elicits greater improvements in inhibitory executive function (superior) than low‐intensity exercise (better). Excessive workload (e.g., intensity and/or duration)‐induced exhaustion may relate to both impairments of dCA and inhibitory executive function.

### 2.5 Dynamic cerebral autoregulation and inhibitory executive function responses to long‐term aerobic exercise

The effect of long‐term AE on relationships between dCA and inhibitory executive function remain unresolved. Perry et al. (2019) reported no difference in dCA during forced ABP oscillations via repeated squat manoeuvres between endurance‐trained and sedentary individuals. In contrast, Drapeau et al. (2019) observed that 6 weeks of HIIE impaired dCA during forced ABP oscillations via repeated squat manoeuvres, despite no change in MCAv. Labrecque et al. (2017) further showed that higher ${\dot{V}}_{O_{2}\max}$ is associated with greater TFA gain and smaller phase during LF forced ABP oscillations via repeated squat manoeuvres, suggesting an inverse relationship between dCA efficiency and cardiorespiratory fitness. Conversely, Liu et al. (2023) found that 12 weeks of HIIE training enhanced inhibitory executive function, consistent with broader evidence that higher fitness levels are associated with superior inhibitory executive function (Dupuy et al., 2015). Together, these findings highlight a complex, and at times contradictory, interaction between vascular regulation, cognitive performance and fitness level, underscoring the need for longitudinal mechanistic studies.

This apparent acute‐to‐chronic discrepancy suggests that the mechanisms underpinning the cognitive benefits of AE may differ, at least in part, between acute and long‐term adaptations. Acute bouts of AE – particularly at higher intensities – may transiently challenge dCA and coincide with reductions in inhibitory executive function, likely reflecting momentary constraints on cerebrovascular reserve. In contrast, long‐term AE appears capable of improving inhibitory executive function even in the presence of unchanged or modestly impaired dCA metrics, suggesting that longer‐term adaptations may partially ‘decouple’ cognitive outcomes from acute cerebrovascular control. Potential mechanisms that may underlie this divergence include long‐term AE‐induced improvements in baseline cerebrovascular health, enhanced NVC, increased cerebral metabolic efficiency, structural adaptations such as angiogenesis, and upregulation of neurotrophic and anti‐OXINOS signalling pathways (Calverley et al., 2020). Collectively, these long‐term adaptations may buffer the cognitive system against transient haemodynamic perturbations observed during acute AE, thereby reconciling improved inhibitory executive function with seemingly unchanged dCA.

## 3 ENERGY CONSUMPTION

Insufficient physical activity remains a global health concern, largely due to lifestyle and time constraints, despite clear evidence that exercise benefits brain and body health (Guthold et al., 2018). Against this background, as well as modifiable behaviours to modify energy expenditure, attention should also be directed toward energy consumption – that is, dietary behaviour – as a feasible and impactful approach to promoting health. Because eating is a routine daily activity, small but consistent choices, such as avoiding meal skipping and prioritising meal quality, may critically influence both dCA and inhibitory executive function.

Regular breakfast consumption has been linked not only to higher Intelligence Quotient (Liu et al., 2013) but also to reduced stroke risk (Kubota et al., 2016). In acute responses, some studies suggest improved cognitive performance, including inhibitory executive function, after breakfast (Galioto & Spitznagel, 2016). Consistent with this, we reported that TFA gain in the LF range during spontaneous ABP fluctuations was lower 2 h after breakfast versus breakfast skipping, suggesting strengthened dCA (Tsukamoto et al., 2021). Given that stroke incidence peaks in the mid‐to‐late morning, coinciding with increases in blood pressure (Sloan et al., 1992), enhanced morning dCA may be especially neuroprotective.

Meal quality also matters. Cognitive function is highly sensitive to blood glucose fluctuations (Lamport et al., 2009). Post‐prandial hyperglycaemia – or ‘spikes’ induced by high‐glycaemic index (GI) meals such as white bread or cornflakes – impairs both inhibitory executive function (Nilsson et al., 2012) and dCA (Figure 4), reflected by smaller TFA phase values in the VLF range during spontaneous ABP fluctuations (Tsukamoto et al., 2021). These effects typically emerge 2–3 h after eating, suggesting that post‐prandial instability in glucose regulation compromises both vascular and cognitive resilience. Moreover, meal timing can exert lasting influences through the ‘second‐meal effect’, whereby breakfast composition modulates post‐lunch glycaemic responses. Skipping breakfast (Nakamura et al., 2008) or consuming a high‐GI breakfast (Liljeberg et al., 1999) exacerbates post‐lunch hyperglycaemia, which in turn impairs inhibitory executive function (Lamport et al., 2011). Thus, breakfast may set the physiological tone for CBF regulation and cognition across the day, making it arguably the most important meal for brain health.

**FIGURE 4:**
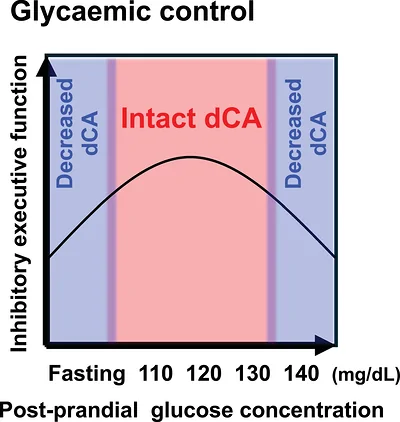
Relationship between dynamic cerebral autoregulation (dCA) and inhibitory executive function in response to glycaemic control. Low‐glycaemic index (GI) meals preserve both dCA and inhibitory executive function, whereas meal skipping or high‐GI meals (post‐prandial hyperglycaemia) impair them.

Given that the second‐meal effect can be observed after skipping lunch or dinner, future studies should examine the effects of other forms of meal skipping, including time‐restricted feeding (TRF), on CBF regulation and inhibitory executive function. For example, early phases of fasting may exacerbate cerebrovascular stress through transient hypoglycaemia/hyperketonaemia (Gibbons et al., 2023) and heightened sympathetic activation (Mesnage et al., 2025), although medium‐chain triglyceride‐induced hyperketonaemia may be associated with an improvement in inhibitory executive function (Yuuki et al., 2026). In contrast, short‐term ketone monoester supplementation may enhance brain health via improved cerebral bioenergetics and endothelial function (Walsh et al., 2021); however, whether ketone production during sustained TRF is sufficient to offset early haemodynamic challenges or restore dCA remains unknown.

Beyond carbohydrates, dietary fat quality may also influence brain function. A single high‐saturated‐fat meal decreases TFA phase and increases gain at VLF forced oscillation, indicating acute dCA impairment due to post‐prandial hyperlipidaemia (Marley et al., 2025). While the direct impact of such meals on inhibitory executive function remains poorly studied, long‐term high‐fat diets have been associated with cognitive decline (Yeomans, 2017). If acute post‐prandial hyperlipidaemia also reduces inhibitory executive function in combination with dCA impairment, then fat quality should be considered as critically as carbohydrate quality when evaluating the effects of diet on cerebrovascular and cognitive health.

Interestingly, the negative effects of post‐prandial hyperglycaemia and hyperlipidaemia on dCA were observed 2–3 h after eating. In terms of blood glucose, this ‘temporal dissociation’ is consistent with a delayed vascular insult rather than a direct glucose‐mediated effect. From a mechanistic perspective, we have proposed that elevated systemic OXINOS represents a potential unifying proximal trigger (Bailey, 2010; Marley et al., 2025, 2017). This response typically coincides with the 2–4 h postprandial peak in circulating triglycerides and lipid‐rich remnants, which increases the availability of oxidisable substrates capable of impairing endothelial and cerebrovascular function. We also acknowledge that insulin‐related mechanisms may contribute to these delayed effects (Tsukamoto et al., 2021). Hyperinsulinaemia has been shown to influence vascular tone, sympathetic activity and endothelial signalling independently of blood glucose (Anderson et al., 1991; Cleland et al., 1998; Sauter et al., 1983), and may therefore modulate both dCA and inhibitory executive function during the postprandial period.

Taken together, converging evidence suggests that meal timing and quality – particularly breakfast – shape coordinated responses of dCA and inhibitory executive function, paralleling the effects of exhaustive AE. These findings underscore the importance of dietary behaviour as a modifiable lifestyle factor influencing cerebrovascular stability and cognition and highlight the need for future work to unravel the precise physiological mechanisms. In addition, chronological meal timing (e.g., late dinner or midnight snacking) and sleep quality/timing represent important, yet underexplored, modifiable behaviours that may influence dCA and inhibitory executive function via altered glucose and lipid handling, heightened nocturnal sympathetic activity, and disrupted endothelial signalling (Chellappa et al., 2025; Morris et al., 2016; Nakashima et al., 2022; Poggiogalle et al., 2018) and are also closely linked to exercise and dietary habits.

## 4 IMPLICATIONS

Although this review primarily focuses on CBF regulation and dCA, other physiological and biochemical pathways may also contribute to the link between modifiable behaviours and inhibitory executive function. AE and dietary patterns are known to influence oxidative stress, the release of exerkines, and angiogenic signalling, all of which may affect neurovascular function and cognitive performance. These mechanisms may act in parallel with, or independently from, changes in dCA, potentially explaining why improvements in inhibitory executive function can occur. While the current literature does not establish a causal relationship between dCA and inhibitory executive function, identifying these pathways may provide targets for future mechanistic studies aimed at clarifying causality.

While energy expenditure and energy consumption have each been shown to influence dCA, their combined effects remain poorly understood. In real‐world settings, prolonged AE is often accompanied by carbohydrate fuelling, which may modify cerebrovascular and cognitive responses through interactions between metabolic availability and cerebral perfusion regulation. During prolonged AE, inadequate substrate availability – particularly carbohydrate – may exacerbate cerebrovascular and cognitive strain through competing metabolic demands, altered glycaemic control and heightened sympathetic activation. Conversely, appropriate fuelling may help preserve cerebrovascular reserve, attenuate stress responses, and stabilise the operating conditions under which dCA and inhibitory executive function are maintained. Future studies should therefore examine how the balance between energy expenditure and energy consumption influences dCA and inhibitory executive function under ecologically valid conditions.

Although much of the available evidence linking dCA, inhibitory executive function and modifiable lifestyle factors is derived from otherwise healthy populations, these interactions may be particularly relevant in ageing and clinical populations. For example, individuals with diabetes – who are highly sensitive to dietary composition and glyceamic regulation – and those with heart failure with preserved ejection fraction (HFpEF), who commonly exhibit exercise intolerance, may exhibit altered dCA–inhibitory executive function crosstalk at baseline or in response to changes in diet and exercise. However, direct evidence in these populations remains limited, highlighting an important area for future research.

## 5 CONCLUSION

An increase in AE intensity generally exerts beneficial effects on inhibitory control, provided that dCA remains functionally intact. By contrast, when dCA becomes impaired – such as during exhaustive AE – inhibitory executive function may be negatively impacted, suggesting that dCA serves as a physiological ‘gatekeeper’ for exercise‐induced acute cognitive benefits.

Similarly, dietary behaviours exert acute influences on both dCA and inhibitory executive function. Breakfast skipping and post‐prandial hyperglycaemia, in particular, have been shown to diminish dCA responsiveness while concurrently impairing inhibitory executive function. Post‐prandial hyperlipidaemia may also contribute to dCA impairment, though its relationship to cognition remains less well defined.

Taken together, these findings support the hypothesis that impairments in dCA represent a shared physiological mechanism through which both energy expenditure and energy consumption can acutely compromise inhibitory executive function. Although causality between dCA and inhibitory executive performance is not yet fully established, the evidence suggests that dCA integrity plays a critical role in buffering the brain against transient haemodynamic and metabolic challenges.

Thus, preserving dCA during modifiable lifestyle behaviours – whether through optimised exercise prescriptions or improved meal timing and quality – may be fundamental to maintaining inhibitory executive function. Future research should aim to clarify the causal pathways linking dCA to cognition, identify thresholds of behavioural stressors beyond which autoregulatory control is compromised, and explore interventions that strengthen dCA to promote brain health.

## AUTHOR CONTRIBUTIONS

Hayato Tsukamoto and Damian M. Bailey conceptualised this review manuscript. Hayato Tsukamoto and Damian M. Bailey drafted and revised it critically for important intellectual content. Both authors approved the final version of the manuscript, agree to be accountable for all aspects of the work, and will ensure that any questions concerning the accuracy or integrity of any part of this work are appropriately investigated and resolved. Both authors designated as authors qualify for authorship, and all those who qualify for authorship are listed.

## CONFLICT OF INTEREST

Hayato Tsukamoto declares no conflict of interest. Damian M. Bailey is Editor‐in‐Chief of *Experimental Physiology*, Chair of the Life Sciences Working Group, member of the Human Spaceflight and Exploration Science Advisory Committee to the European Space Agency and member of the Space Exploration Advisory Committee to the UK and Swedish National Space Agencies. Damian M. Bailey is also affiliated to Bexorg, Inc. (USA), focused on the technological development of novel biomarkers of cerebral bioenergetic function and structural damage in humans.
